# Exploitation of Marine-Derived Robust Biological Molecules to Manage Inflammatory Bowel Disease

**DOI:** 10.3390/md19040196

**Published:** 2021-03-30

**Authors:** Muhammad Bilal, Leonardo Vieira Nunes, Marco Thúlio Saviatto Duarte, Luiz Fernando Romanholo Ferreira, Renato Nery Soriano, Hafiz M. N. Iqbal

**Affiliations:** 1 School of Life Science and Food Engineering, Huaiyin Institute of Technology, Huaian 223003, China; 2 Department of Medicine, Federal University of Juiz de Fora, Juiz de Fora-MG 36036-900, Brazil; leonardo.nunes@estudante.ufjf.br; 3 Department of Medicine, Federal University of Juiz de Fora, Governador Valadares-MG 35010-180, Brazil; marco.saviatto@estudante.ufjf.br; 4 Graduate Program in Process Engineering, Tiradentes University (UNIT), Av. Murilo Dantas, 300, Farolândia, Aracaju-Sergipe 49032-490, Brazil; luiz.fernando@souunit.com.br; 5 Institute of Technology and Research (ITP), Tiradentes University (UNIT), Av. Murilo Dantas, 300, Farolândia, Aracaju-Sergipe 49032-490, Brazil; 6 Division of Physiology and Biophysics, Department of Basic Life Sciences, Federal University of Juiz de Fora, Governador Valadares-MG 35010-180, Brazil; renato.soriano@ufjf.edu.br; 7 School of Engineering and Sciences, Tecnologico de Monterrey, Monterrey 64849, Mexico

**Keywords:** algal biome, polysaccharides, bioactive entities, engineered cues, therapeutic attributes, inflammatory bowel disease

## Abstract

Naturally occurring biological entities with extractable and tunable structural and functional characteristics, along with therapeutic attributes, are of supreme interest for strengthening the twenty-first-century biomedical settings. Irrespective of ongoing technological and clinical advancement, traditional medicinal practices to address and manage inflammatory bowel disease (IBD) are inefficient and the effect of the administered therapeutic cues is limited. The reasonable immune response or invasion should also be circumvented for successful clinical translation of engineered cues as highly efficient and robust bioactive entities. In this context, research is underway worldwide, and researchers have redirected or regained their interests in valorizing the naturally occurring biological entities/resources, for example, algal biome so-called “treasure of untouched or underexploited sources”. Algal biome from the marine environment is an immense source of excellence that has also been demonstrated as a source of bioactive compounds with unique chemical, structural, and functional features. Moreover, the molecular modeling and synthesis of new drugs based on marine-derived therapeutic and biological cues can show greater efficacy and specificity for the therapeutics. Herein, an effort has been made to cover the existing literature gap on the exploitation of naturally occurring biological entities/resources to address and efficiently manage IBD. Following a brief background study, a focus was given to design characteristics, performance evaluation of engineered cues, and point-of-care IBD therapeutics of diverse bioactive compounds from the algal biome. Noteworthy potentialities of marine-derived biologically active compounds have also been spotlighted to underlying the impact role of bio-active elements with the related pathways. The current review is also focused on the applied standpoint and clinical translation of marine-derived bioactive compounds. Furthermore, a detailed overview of clinical applications and future perspectives are also given in this review.

## 1. Introduction

**Figure 1:**
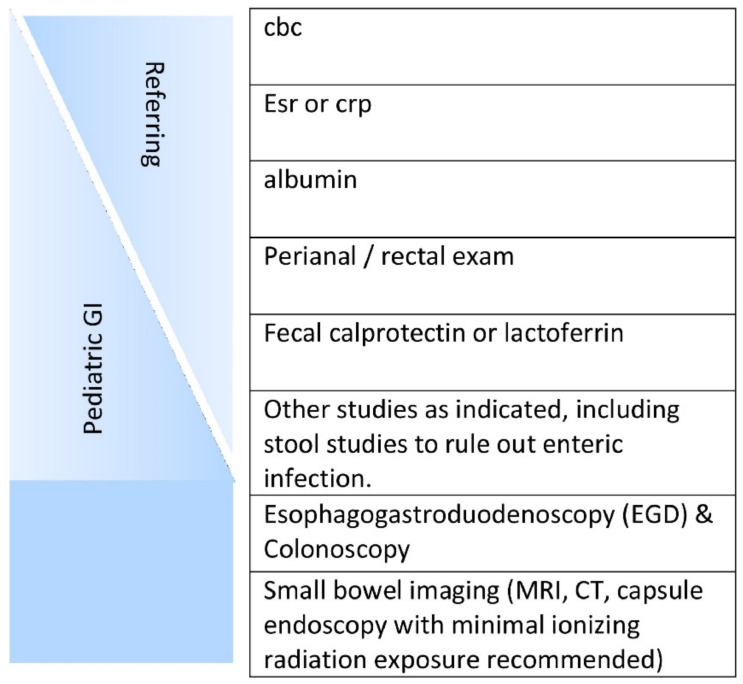
Initial workup of a patient suspected of having inflammatory bowel disease (IBD). The initial workup is ideally started by the referring physician, with the subspecialist performing anything missing plus endoscopies and small bowel assessment(s) [1]. License Number: 5022881429496. Abbreviations: CBC (Complete Blood Count), CRP (C-reactive protein), ESR (Erythrocyte Sedimentation Rate), MRI (Magnetic resonance imaging), and CT (computed tomography).

Inflammatory bowel disease (IBD) is a chronic inflammation of the gastrointestinal tract (GIT) that occurs due to the dysregulation of the immune system. Although the explicit etiology and underlying remain uncertain, both environmental and genetic factors are involved in immune dysregulation. Historically, IBD has been categorized into Crohn’s disease (CD) and ulcerative colitis (UC). Both diseases show heterogeneous pathological and clinical features and can be distinguished by their location, nature and characteristics of inflammation. More specifically, the laboatory analysis and careful stool evaluation are considered initial measures to diagnose the patient who is suspected to have IBD (Figure 1) [1]. Crohn’s disease is a deeper transmural inflammation affecting any segment of the GI tract from the mouth to anus, whereas ulcerative colitis is a chronic inflammatory disease that attacks the colonic mucosa [1]. Approximately, 25% of UC patients require hospitalization for acute severe ulcerative colitis (ASUC) at any phase during this complication, leading to colectomy in 40% of patients [2,3].

**Figure 2:**
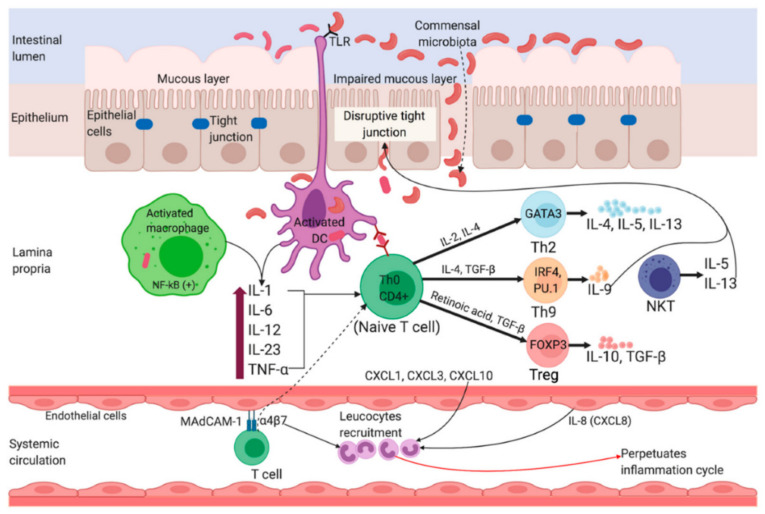
Pathophysiology of Ulcerative Colitis. Impairment of tight junctions and the mucous layer leads to increased permeability of the intestinal epithelium, resulting in more uptake of luminal antigens. Antigen presenting cells (APC) become activated upon recognizing non-pathogenic bacteria (commensal microbiota) through Toll-like receptors (TLRs). Activated APC initiate differentiation of naïve CD4+ T-cells into Th-2 effector cells (which produce pro-inflammatory cytokines such as TNF-α, IL-5, IL-6, and IL-13). TNF-α and IL-1 activate nuclear factor κB (NF-κB) pathway, which facilitate expression of pro-inflammatory and cell survival genes. Binding of integrin-α4β7 bearing T cells to the mucosal adhesion molecule MAdCAM-1 facilitate entry of more T cells into the lamina propria. Recruitment of circulating leucocytes due to the upregulation of inflammatory chemokines (chemokine ligands: CXCL1, CXCL3, CXCL8 and CXCL10) perpetuates the inflammatory cycle. MAdCAM-1, mucosal addressin cell adhesion molecule-1; IL, interleukin; TNF-α, tumor necrosis factor-alpha; TGF-β, transforming growth factor-beta; NKT, natural killer T; DC, dendritic cell; Th, T helper; GATA3, GATA binding protein 3; IRF4, interferon regulatory factor 4; PU.1, purine-rich PU-box binding protein; FOXP3, Forkhead box protein 3. Reprinted from Ref. [4] with permission under the Creative Commons Attribution (CC BY) license. Copyright © 2020 the authors. Licensee MDPI, Basel, Switzerland.

**Figure 3:**
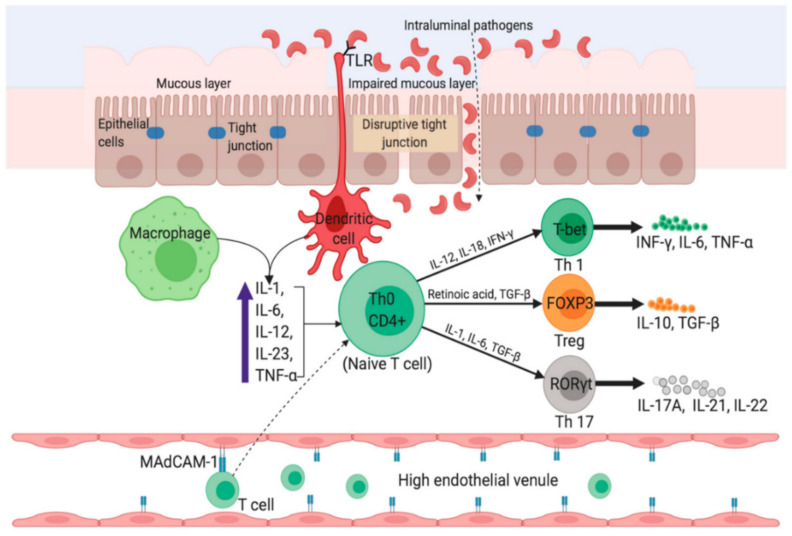
Pathophysiology in Crohn’s disease. The uptake of luminal microflora stimulates APCs (e.g., dendritic cells and macrophages) which in turn produce proinflammatory cytokines such as TNF-α, IL-6, and IL-23. Activated APCs facilitate subsequent differentiation of naïve CD4^+^ T-cells into Th1 and Th17 via expression of master transcription factors. Inside the high endothelial venule, binding of α4β7-bearing lymphocytes to MAdCAM-1 causes entry of more T cells into the lamina propria. IFN-γ, interferon-gamma; FOXP3, Forkhead box protein 3; RORγt, retinoic acid receptor-related orphan nuclear receptor gamma. Reprinted from Ref. [4] with permission under the Creative Commons Attribution (CC BY) license. Copyright © 2020 the authors. Licensee MDPI, Basel, Switzerland.

The origin and disease progression of UC and CD are significantly different from each other. The changes in microbial diversity of lumen, impaired barrier functions of mucus and epithelial layer through interrupting tight junctions are strongly associated with the UC pathogenesis. Figure 2 portrays a graphical representation of the pathophysiology of UC [4]. Though individuals with UC show a great percentage of Enterobacteriaceae Gamma-proteobacteria [5], and sulfite-reducing bacteria [6], and minimum Firmicutes diversity, such changes are intestinal inflammation-mediated or vice versa remains debatable. In the case of CD, inflammation of the small bowel results in an increased concentration of pro-inflammatory cytokines, like IL-17A, and IFN-γ [7]. Furthermore, Th17 cell-derived IL-17 in turn favors the Th-1 response [8]. IL-6, IL-23, and TGF-β secreted by antigen-presenting and innate immune cells influence the IL-17 pathway (Figure 3) [4].

In 2017, about 6.8 million cases of IBD were documented worldwide [9]. Over 1.6 million, 85,000, 250,000 and 260,000 people are affected by IBD in Australia, the USA, the UK and China, respectively [10,11,12]. IBD remains dominant in western countries in the last few decades because of the higher prevalence and incidence rates than in the developing world. Nevertheless, the incidence of IBD has now intensified dramatically in several Asian countries [12] with a consistently increasing trend, mainly in China, Japan, Hong Kong, and Korea [13]. Due to lacking national registries in many African, Asian, and Latin American nations, there is very scarce information regarding the occurrence and prevalence of IBD. 

IBD has been reported to occur at any age, however, the peak incidence appears in early adulthood and adolescence [14,15,16]. Symptoms associated with IBD can be unpredicted and highly variable. Children may exhibit inimitable physical examination findings along with several upper or lower GI manifestations. There might be only a few symptoms in some cases, with inexplicable weight loss and growth retardation. It is imperative to distinguish that IBD is not irritable bowel syndrome (IBS). Though both diseases may present identical symptoms, only IBD results in stunted height, growth retardation, ostomies, surgeries, and many other undesirable outcomes or risks. Besides the systemic symptoms, like fatigue, fever, mouth sores, uveitis, arthralgia, and nail clubbing, IBD is also related to a variety of extra-intestinal symptoms. These manifestations appear in approximately 25–35% of individuals with IBD particularly at a young age [17,18].

The treatment protocols being practiced for the IBD involve either medication therapy or surgery [19]. The preferred choice of medication therapy in the case of IBD is the treatment with the use of anti-inflammatory drugs, such as aminosalicylates and corticosteroids. The second line medication for IBD encompasses using immunosuppressants to halt the immune response, responsible for un-regulated inflammation. In addition to immunosuppressants, tumor necrosis factor-α (TNF-α) inhibitors, or biologics works by neutralizing immune system protein. In Crohn’s disease, where the infection is a concern, antibiotics can be used to reduce the chances of infection. On the other hand, surgery may be an option in severe cases when all these therapeutic options do not work. However, treatment protocols are also being practiced culminating devastating colorectal cancer, but no treatment is required for benevolent stage cancer. In the case of metastatic invasion, surgery can be opted to eradicate malignant tumors and lymph nodes [20].

The marine environment has been demonstrated as a prolific source of bio-active compounds with unique chemical, structural and functional features. Furthermore, the molecular modeling and synthesis of new drugs based on marine-derived therapeutic and biological cues might present greater efficacy and specificity for the therapeutics (Table 1). A vast number of compounds have been derived and identified from marine sources that exhibit a noteworthy role to circumvent the reactive oxygen species (ROS) generation, show anti-inflammatory effects, and hinder various metabolic pathways. The current review is focused on marine-derived bioactive compounds to treat and manage IBD. Furthermore, a detailed overview of clinical applications and future perspectives are also given in this review.

## 2. Marine-Derived Bioactive Compounds against Inflammatory Bowel Diseases (IBD)

### 2.1. Chitosan-Structural Properties and Potential Therapy of IBD

Chitosan is a linear polysaccharide obtained from deacetylation of chitin, it has a cationic character because of its primary amino groups, which provides it with properties such as controlled drug release, mucoadhesion, in situ gelations, transfection and increased permeation [44,45]. It is an important constituent part of the exoskeleton of arthropods, fungi, and crustaceans, which are the main marine source [46,47]. Among its various properties and applications, those that stand out most are antimicrobial, antioxidant, anticancer and anti-inflammatory [32,33,34,35,36,37].

The mucoadhesive property of chitosan has been a key point of its application in the treatment of IBD (Table 2) and has also been indicated in a study with patients with IBD [48,49]. For rectal use, this characteristic allows a prolonged local retention time of the drugs. In line with this idea, a chitosan-based hydrogel was able to enhance the efficacy of rectal administration of sulfasalazine (SSZ) in a mice model of ulcerative colitis. The mucoadhesive drug delivery system was more therapeutic than the conventional oral treatment and reduced the plasma concentration of a potentially toxic by-product of the drug [50]. Besides its excellent mucosa adhesion, chitosan polymers are widely employed in targeted drug delivery systems, providing better efficacy results in oral treatments. Pillay et al. were able to develop Stimuli-Synchronized-Matrix (SSM) for colonic delivery, defined by the space, of mesalamine (5-amino-salicylic acid or 5-ASA), the therapeutic metabolite of SSZ, employing chitosan in a polysaccharide matrix coated with an alloy layer [51]. Their SSM was both time and pH-independent, while provided a responsive release of the chemical in the presence of colonic enzymes. These characteristics also allowed the minimization of variations of the plasma concentration and reduced the systemic presence of 5-ASA. A similar approach was used in bioadhesive chitosan pallets and coated beads to increase the topical delivery of 5-ASA [52,53,54]. Mesalamine has also been loaded in N-succinyl-chitosan microparticles, improving its therapeutic results [54].

Additional research has been conducted to ameliorate the pharmacokinetics of the conventional treatment for IBD. A microgel based on oxidized sodium alginate and water-soluble chitosan demonstrated in vitro potential application as a carrier for mesalamine [55]. A different formulation of chitosan and alginate composite microparticles associated with an enteric coat was capable of effectively delivering 5-ASA and curcumin in a colitis rat model [58]. Another preparation combined a cellulose-derived polymer with pectin and chitosan in matrix tablets of 5-ASA to provide desirable changes in its physicochemical characteristics and drug release profiles [56]. An additional antibacterial effect was reached by preparing mesalamine tablets with a chitosan-ethylenediaminediacetic acid disodium (CH-EDTA) conjugate coating [57]. Research has also been conducted to improve the pharmacokinetics of immunosuppressive drugs. Some strategies, such as loading chitosan modified lipid nanoparticles (NPs) with dexamethasone and preparing azathioprine-loaded chitosan beads have shown promising results in targeted and sensitive drug delivery at the colitis site [59,60]. Dexamethasone microcrystals coated with chitosan, alginate, and an enteric coat multilayers also exhibited significant therapeutic effects in mice [61]. Another corticoid, prednisolone, was loaded with inulin, a naturally occurring polysaccharide, in beads and microparticles coated with calcium (Ca)-alginate core and a chitosan coating. The resulting formulations were tested in vitro and were deemed suitable to be used to deliver substances to the colon [62].

**Figure 4:**
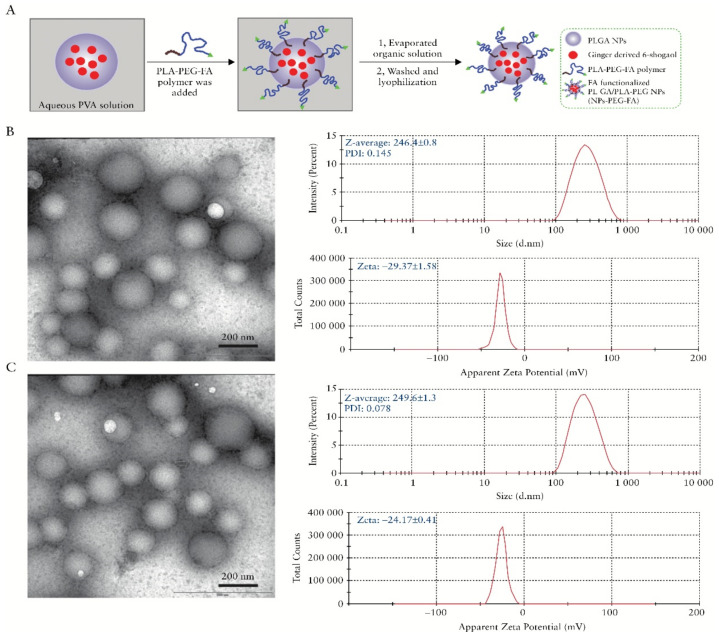
Preparation and characterization of 6-shogaol loaded polymeric nanoparticles, (**A**) Schematic illustration of process through which PLGA/PLA-PEG-FA nanoparticles [NPs-PEG-FA] were fabricated using a versatile single-step surface-functionalising technique, (**B**) The morphology of PLGA/PLA-PEG nanoparticles [NPs-PEG] was characterised by transmission electron microscopy [TEM], and their size and zeta potential were measured by dynamic light scattering [DLS] using a Malvern Zetasizer Nano ZS90 Apparatus, and (**C**) The morphology, size, and zeta potential of PLGA/PLA-PEG-FA [NPs-PEG-FA] were characterized. Reprinted from Ref. [66] with permission from Oxford University Press. Copyright © 2017, Oxford University Press. License Number: 5022890402536. Abbreviations: PVA (Polyvinyl alcohol), PLGA (poly(lactic-co-glycolic acid)), PLA (Polylactic acid), PEG (Polyethylene glycol), FA (Folic acid), and NPs (Nanoparticles).

The possibility of producing hydrogels with chitosan to obtain a sustained release of a drug in the intestine is another relevant aspect for its application in the treatment of IBD. A biodegradable and reversible polyelectrolyte complex (PEC) of poly acrylic-acid (PAA) and chitosan was engineered to be used as a swellable hydrogel colonic delivery system for topical treatment of IBD with pentosan polysulfate (PP). However, an enteric coat was still needed to protect the proposed formulation from the gastric environment [63]. Resveratrol, a polyphenol present in red wine with anti-inflammatory properties, was also successfully loaded in nanostructured chitosan-based hydrogel and had its pharmacokinetics improved [64]. Two similar studies demonstrated the use of a chitosan and alginate hydrogel to encapsulate nanoparticles loaded with curcumin and 6-shogaol, a biologic compound found in ginger. The formulations significantly alleviated the colitis symptoms and quickened wound repair in mice (Figure 4) [65,66]. Interesting use of other chitosan hydrogel was in the design of an antibody functionalized nanoparticles-releasing hydrogel. The authors developed nanoparticles prepared with single-chain CD98 antibodies on their surface, for carrying inside of it CD98 small interfering RNAs (siCD98). It was already known that the overexpression of CD98 in the colonic epithelial cells and macrophages was associated with the development and progression of IBD. By using this strategy, the authors were able to downregulate CD98 and efficiently diminished the manifestations of IBD in vitro and in vivo [67]. This idea of using oligonucleotides to inhibit the synthesis of pro-inflammatory molecules has been recently employed to reduce TNF-α production in a mice colitis model. Xu et al. [68] loaded a chitosan-alginate hydrogel with phosphorothioated antisense oligodeoxyribonucleotide of TNF-α (PS-ATNF-α) and reportedly inhibited the molecule at both the protein and mRNA levels [68].

A different approach for targeted delivery of anti-inflammatory substances to macrophages was reported by Chen et al. [69]. The researchers used chitosan associated with alginate and tripolyphosphate (TPP) to form colloidal particles with the drug tylophorine malate (NK007) and to incorporate it inside glucan mannan particles (GMPs). The formulation was capable of specifically delivering the drug to macrophages and effectively cured colitis in the mice model after being administered orally [69]. Chitosan was also used in association with alginate to form gastroprotective microparticles capable of releasing in the small intestine and colon nanoparticles of AvrA, an anti-inflammatory and anti-apoptotic bacterial protein. The authors reported that the formulation diminished clinical and histological scores of inflammations in a colitis model. The encapsulation with alginate and chitosan allowed the drug to be administered orally, instead of transrectally, which was undesired and restricted the delivery to the distal portion of the colon [70]. This association was also applied to develop alginate/chitosan-coated pellets intended for the colon delivery of rutin, a flavonoid with antioxidant and anti-inflammatory effects. The results were promising dissolution profiles and great stability for rutin, which could be a valuable alternative for mild-to-moderate IBD therapy [71].

An analogous approach was applied to the development of chitosan-alginate microspheres as a carrier for icariin, a type of flavonoid, to reduce colonic injury and inflammatory response in rats [72]. Chitosan-alginate microspheres have also been employed to colon deliver a combination of ketoprofen and ascorbic acid, bringing together anti-inflammatory and antioxidant properties [81]. Quercetin, a flavonoid, has been incorporated in a chitosan-xanthan microparticle coated tablet, allowing a sustained and targeted delivery to the target [73]. Alike, a hybrid system made liposomes coated with cross-linked chitosan was proposed to deliver quercetin to the intestine [74]. Another strategy to protect a drug against the gastric environment and control its release within the intestine was the development of carboxymethyl starch (CMS)—chitosan monolithic tablets. A study has employed these tablets as a carrier for diamine oxidase and catalase, two enzymes possibly capable of reducing bowel inflammation [75]. A chitosan-derived polymer-enzyme conjugate was developed as a promising option in the treatment of IBD with superoxide dismutase, an antioxidant enzyme [76]. Also for oral delivery, a nanocarrier based on chitosan and fucoidan was loaded with berberine, an alkaloid capable of promoting tightness of the intestinal epithelial tight junction, and revealed promising results [77]. 

**Figure 5:**
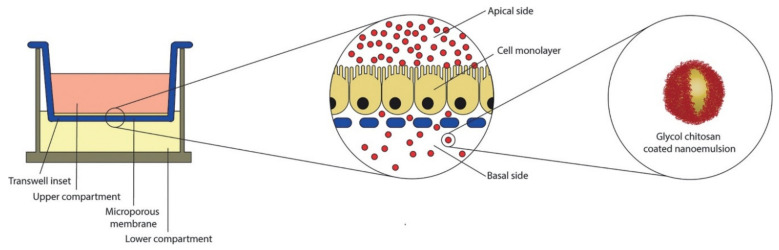
Schematic representation of air liquid interface of human colon carcinoma cell line (CaCo-2) equivalent epithelium in Transwell system. In the central insert, an enlarged view of the cell monolayer grown on the microporous membrane. On the right, the oral nano-delivery system consisting of oil-in-water (O/W) nanoemulsion coated with a thiolated glycol chitosan. Reprinted from Ref. [78] with permission from Elsevier. Copyright © 2018 Elsevier B.V. License Number: 5022890619360.

A different design proposed an oil-in-water nanoemulsion coated with a chitosan-based polysaccharide layer film as a nanocarrier for curcumin, an agent with anti-inflammatory and antioxidant properties but lipophilic and unstable in aqueous solutions. The in vitro tests demonstrated that the formulation was capable of protecting the drug from degradation, evidencing its promising use for oral delivery of similar agents (Figure 5) [78]. A multiple stepwise spinning disk processing (SDP) technique was developed to fabricate a drug delivery system based on a composite diclofenac sodium-chitosan-poly(methyl acrylates) nanoparticulate. This approach allowed scale-up manufacturing of the nanoparticulate. Additionally, the drug uptake noticed was three times higher than the control drug solution, with no evident toxicity [79].

A more complex approach employed chitosan nanoparticles as a delivery vector for gene therapy in a colitis mice model. The authors developed a very cost-effective way of constructing a carrier for a plasmid of Single Ig-domain containing IL-1 receptor-related molecule (SIGIRR), a subtype of the IL-1R family, capable of attenuate colonic tissue inflammation as a result of the inhibition of TLR4/NF-κB overactivation. The study suggested a possible new gene therapy for IBD [80]. Along with the possibility of improving the pharmacokinetics of other compounds, Chitosan oligosaccharide (COS), the major degradation product of chitosan, has also been shown biological activity [82]. For this reason, it has been pointed out as a potential compound to be used in colitis-associated colorectal cancer (CRC) chemoprevention, due to its activation of AMP-activated protein kinase (AMPK) and inhibition of the NF-κB and mTOR signaling pathways in intestinal epithelial cells (IEC) [83,84,85,86]. 

Another relevant application of chitosan is in tissue engineering. Its antimicrobial effect and biodegradability have made it possible for the compound to be used as a bioscaffold for colorectal tissue engineering. A study has shown that a 3-layer chitosan hydrogel patch revealed good wound healing, effective regulation of the inflammation, and an integral regeneration of the colonic wall, along with the smooth cell layer. The latter was achievable due to the soft gel layers on each side, which ensured the colonization of cells and the formation of neo-tissue [87]. In a different approach, an association of chitosan-based hydrogel and stromal vascular fraction from adipose tissue has successfully replaced a circular colonic wall section [88]. Chitosan grafts could be useful for severe cases of IBD that require surgical intervention, such as those on which cancer develops.

### 2.2. Hyaluronic Acid-Physicochemical Attributes and Potential Therapy of IBD

Hyaluronic acid (HA) is the only GAG that is not sulfated and not bound to proteins and it’s formed by units of disaccharides N-acetyl-d-glucosamine (GalNAc) and d-glucuronic acid (GlcA) [89,90]. HA is a crucial component of the extracellular matrix and performs several functions like cell signaling mediation, morphogenesis, damage repair, and matrix organization [91,92]. HA is found in almost all tissue in humans and also in other vertebrates, can be extracted from marine sources like a shark, stingray, eyeball, liver of swordfish, mollusk bivalves, and tuna [93,94,95,96]. It has numerous applications in biotechnology, regenerative medicine, drug delivery vehicles, development of new biomaterials, and other biomedicals and pharmaceutical applications by cause of their properties as biocompatibility, viscoelasticity, lubricity, and immunostimulatory [93,97,98]. HA has been studied as a potential therapeutic agent against several diseases due to its antioxidant, anti-inflammatory, and anticoagulant biological activities [27,28,29,30,31]. 

HA has potentially useful biological activities against IBD, and studies have shown that sodium hyaluronate, a derivative of HA, has beneficial effects in the treatment for IBD. To investigate whether supplementation of the lining of the colon mucosa with sodium hyaluronate may be a possible and effective alternative treatment for IBD, a clinical study recruited 21 individuals with distal UC and applied 60 mL of sodium hyaluronate gel (IBD98E) once a day for 28 days. 38.1% of patients achieved clinical remission and 47.6% achieved endoscopic remission, showing that the local application of IBD98E enhances endoscopic and clinical outcomes in subjects with active distal UC [99]. However, this study did not have a placebo-controlled group, therefore, the results need to be analyzed with caution. Another study demonstrated the improvement of mucosal healing by quickening intestinal epithelial repair with HA therapy in vitro and in vivo [100]. HA can also be an option to improve the effectiveness of conventional treatments like mesalamine (5-ASA), combined therapy with HA and 5-ASA was capable to accelerate wound repair and diminish inflammatory reaction in rat colitis [101]. 

**Figure 6:**
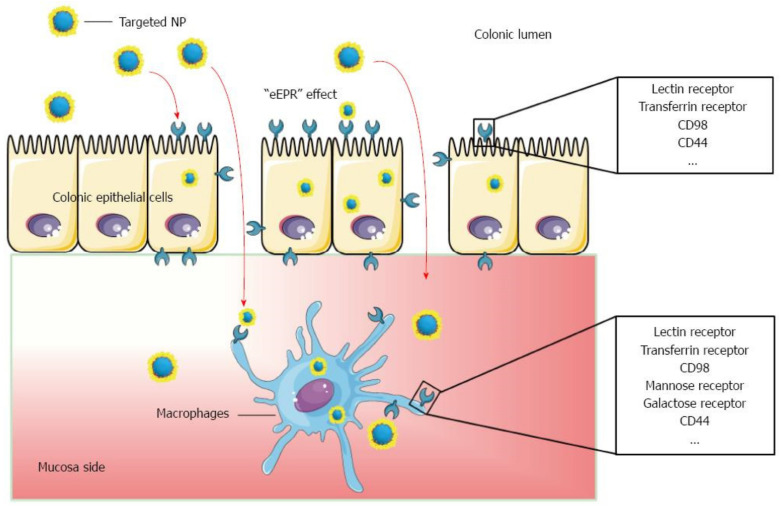
Schematic illustration of orally administered cell-specific nanotherapeutics for IBD. Reprinted from Ref. [111] with permission under the Creative Commons Attribution Non Commercial (CC BY-NC 4.0) license. Copyright ©The Author(s) 2016. Published by Baishideng Publishing Group Inc.

Another potential application of HA was reported in a novel study based on the synthesis of methylcellulose (MC) and HA-coated thermo-responsive hydrogel for successful targeted rectal delivery against IBD [102]. The gelling behavior of the hydrogel was improved due to the in situ gelling capability of HA [103]. Moreover, other remarkable features of HA like the slow and prolonged controlled release, non-toxic, stability, and entero-protection were also observed in the mice intestinal model via applicating final formulated hydrogel. It was evident that the HA-MC hydrogel can be employed in safe and inexpensive systems for rectal delivery of substances in IBD [104]. IBD is a risk factor for the development of colorectal cancer and some studies also pointed out applications of HA in these cancer treatments [105,106,107,108]. In the last years, nanoparticles (NPs) have been identified with promising strategies for the diagnosis and treatment of many diseases. In comparison to more traditional approaches, they offer advantages such as nanometer-scale dimension, controlled drug release, targeted drug delivery capacity, lower plasma concentrations, and lessen adverse effects [109,110,111]. Several pieces of research with NPs have been conducted to try to overcome the biggest problems with oral drug administration such as loss of stability in GIT, systemic absorption, risk of side effects, difficulty in transporting sufficient quantities of active drugs, and transporting them to specific target sites [111,112]. In this regard, NPs have been an innovative approach for IBD treatment, and the results have shown that they are much more effective than traditional drug formulation (Figure 6) [111].

Lysine-proline-valine (KPV) is a tripeptide with anti-inflammatory properties, Xiao et al. [113] loaded KPV into HA-functionalized polymeric nanoparticles (HANPs), resulting in an NPs called HA-KPV-NPs. The authors encapsulated the HA-KPV-NPs in a hydrogel (chitosan/alginate) for oral administration against UC in a mouse model and used a group control without HA. The results show that HA-KPV-NPs/Hydrogel system exhibited a much stronger capacity to prevent mucosa injury and downregulate TNF-α compared with group control. Therefore, this study reveals the important role of the HA in the NPs, which was able to penetrate the colitis tissue and allow the KPV to be internalized in the target cells, thereby alleviate UC [113]. In another study that also used a model of UC in mice, HANPs was used to deliver the siRNA of the CD98 transmembrane protein involved in the colon’s innate immune responses, siCD98, in association with a robust anti-inflammatory agent, curcumin. The results of the analyzes showed that cell uptake of drugs in groups treated with HANPs was much higher than those treated with NPs without HA, demonstrating that HANPs could be a good alternative for UC-target therapy [114]. The same group reported in previous studies that surface functionalization with HA increases the cellular uptake efficiency of NPs as a consequence of interactions mediated by receptor [67], which seems to be related to the fact that HA is a ligand of CD44, a membrane glycoprotein, that in UC has increased expression on the surfaces of colon epithelial cells and macrophages [115,116,117]. Accordingly, Vafaei et al. [118] used HANPs to release budesonide in an in vitro model of inflamed CACO-2 cells, the anti-inflammatory effect with HAMPs was much greater compared to cells that received the free drug [118]. Similar to HANPs, Li et al. [119] use HA functionalized porous silicon nanoparticles to unite enzyme-responsive hydrogel and pH-responsive polymer to develop a hierarchical structured and programmable responsive AP@PSi-HA@HPMCAS carrier for efficient local delivery of drugs to sites of inflammation in the intestine in IBD treatment via oral administrations. The vehicle with HA exhibited superior therapeutic efficacy and significantly diminished systemic drug exposure [119]. No clinical studies have been found using HANPs to treat IBD, however, due to wide marine availability and the promising results of experimental studies they have great potential for the developing of alternative therapies capable of overcoming the limitations of traditional oral drug formulation. 

### 2.3. Chondroitin Sulfate-Physicochemical Traits and Potential Therapy of IBD

Chondroitin Sulfate (CS) is a sulfated glycosaminoglycan (GAG) found on cell surfaces and inside the pericellular matrix in the form of proteoglycans from different organisms and is involved with several physiological events [120,121]. CS is a linear polysaccharide formed by the repetition of disaccharide units N-acetyl-d-galactosamine (GalNAc) and d-glucuronic acid (GlcA), which have sulfate groups added by several sulfotransferases in various positions, forming diverse structures with heterogenous characteristics [122,123]. Commercially, the main marine source of CS is shark cartilage but it can also be extracted from octopus, salmon, zebrafish, ray, and squid [96]. CS has biological activity as an immunomodulator, anti-inflammatory, anticancer, antiviral, and anticoagulant [21,22,23,24,25,26]. It has been highly employed in the treatment of osteoarthritis and tissue engineering, as CS hydrogels proved to accelerate wound healing [21,96] and several studies have investigated its use in the management of IBD.

**Figure 7:**
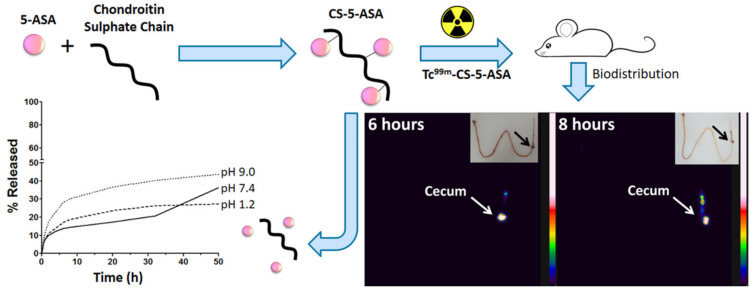
Mesalamine polymeric conjugate for controlled release. Reprinted from Ref. [124] with permission from Elsevier. Copyright © 2017 Elsevier B.V. License Number: 5022891040345.

Similar to chitosan, research has been conducted to employ CS to obtain a controlled release of drugs already in use to treat IBD. Cesar et al. [124] successfully synthesized a polymeric conjugate of 5-ASA and CS capable of improving the biodistribution of the drug and of providing it with a mucoadhesive profile (Figure 7) [124]. The authors suggested that the pharmaceutical could be considered as an alternative therapy for ulcerative colitis [124]. Prednisolone has also been conjugated with CS to improve its delivery to the lower intestines. The result was a nanogel that had its ability to target areas affected by colitis confirmed in vivo [125]. Furthermore, the multi-bioresponsive drug, curcumin, has been used in association with CS. Two studies described the development of curcumin-loaded nanoparticles with their surfaces functionalized with CS. This modification yielded prominently targeted drug delivery to macrophages and was considered a promising therapeutic platform for IBD [65,126]. Additionally, a prospective, observational, follow-up study indicated that IBD patients in remission under CS treatment for osteoarthritis could have a lower incidence of relapses than generally reported in the literature. Although the study had a small sample, it suggested that CS could be applied as a candidate for the treatment of IBD due to its good safety profile and capability of alleviating osteoarthritis-related pain in these patients [127].

### 2.4. Alginate-Physicochemical Attributes and Therapeutic Option for IBD

Alginates are a natural biopolymer composed of 1,4-linked (-l-guluronic acid) (G) and (-d-mannuronic acid) (M) residues to form GG, GM, and MM blocks [128]. *Ascophyllum nodosum, Laminaria hyperborea, Saccharina japonica, Macrocystis pyrifera, Laminaria digitata*, and other species of brown seaweeds are the main source extraction with 17 to 45% of the dry weight composed of alginate [129,130]. It is widely available, biocompatible, non-toxic, low cost a has wide application in food processing, biotechnology, and biomedical industry [131,132]. Some studies have pointed out the biological activities of alginates as an antioxidant, anti-inflammatory, immunomodulating, anticoagulant, and anticancer [38,39,40,41,42,43].

Alginate has non-toxic, non-immunogenic, biocompatible and biodegradable properties [131,133,134], which make it a versatile material for biomedical application as in microspheres, microcapsules, gel beads, hydrogel, film, nanoparticles, pellets, and tablets for drug delivery [133,135]. Several alginate formulations with other marine compounds have been studied in treatments against IBD and some of them were described previously with chitosan. The main applications are in the engineering of drug delivery systems specifically for the colon, which allows its use in the treatment of IBD in a different way than relying solely on pH sensitivity [136]. In this regard, alginate offers a range of microencapsulation through a series of techniques, Samak et al. [137] analyzed alginate microparticles loaded with hydrocortisone hemisuccinate and fabricated via aerosolization and homogenization methods. In vitro experiments were carried out to verify whether the microparticles by each of the techniques were able to suppress the release of the drug in the simulated gastric fluid and promote the release correctly in the simulated intestinal fluid. The results indicated that both methods show potential for producing alginate hydrogel microparticles suitable for colon-specific drug delivery in IBD treatment [137]. A subsequent study confirmed the potential of these techniques for delivering *Nigella sativa* extract using the same in vitro tests [138]. Other studies have investigated alginate formulations to see if they can improve the delivery and effectiveness of drugs used in conventional IBD treatments like corticosteroids) [139,140].

## 3. Concluding Remarks and Outlook

Inflammatory bowel disease continues to result in substantial productivity loss and extensive morbidity worldwide. The devastating consequence of inflammatory bowel disease urgently calls for proper treatments, and thus implicate a considerable financial and economic burden to individuals and the entire health care structure, particularly in emerging countries. Marine-derived bioproducts attract incredible interest and appear a revolutionary therapy for IBD due to their health beneficial properties that are ascribed to the presence of characteristic biologically active functional constituents. This review presents some naturally occurring biological entities/compounds derived from different marine species for the efficient management of IBD. Particular focus has been devoted to characteristic attributes, performance evaluation of engineered cues, and point-of-care IBD therapeutics of diverse bioactive compounds from the algal biome. Although the ratio of early surgery is reduced in IBD-associated patient by introducing biological active substances based therapeutic modalities, many challenges remain. Hence, additional research efforts are required to inspect their bioavailability and efficiency in human and animal models. The exploration of marine-derived bioactive compounds will continue to increase in the future depending on new extraction strategies and novel modes of action. Ideally, intensive future research and new research opportunities for marine bioproducts would lead to a more efficacious and safer way for preventing and management of inflammatory bowel disease.

## Figures and Tables

**Table 1:** Marine-derived compounds and biological activities with therapeutic potential.

Marine-Derived Compound	Sources	Potential Applications and Benefits	References
Chondroitin Sulfate (CS)	Shark cartilage, octopus, salmon, zebrafish, ray, squid	Antiinflammatory, Immunomodulatory, Anticancer, Antiviral, Anticoagulant	[21,22,23,24,25,26]
Hyaluronic acid (HA)	Shark, stingray, eyeball, liver of swordfish, mollusk bivalves, tuna	Anti-inflammatory, Antioxidant, Anticoagulant	[27,28,29,30,31]
Chitosan	Arthropods (crustaceans), fungi	Anti-inflammatory, Antioxidant, Anticancer, Antimicrobial	[32,33,34,35,36,37]
Alginate	Brown seaweeds	Anti-inflammatory, Immunomodulatory Antioxidant, Anticancer, Anticoagulant	[38,39,40,41,42,43]

**Table 2:** Applications of Chitosan in drug delivery systems for IBD treatment.

Strategy	Drug Delivered	References
Hydrogel	Sulfasalazine	[50]
Coated matrix	5-amino-salicylic acid (5-ASA)	[51]
Coated beads	5-amino-salicylic acid (5-ASA)	[53,54]
Microparticles	5-amino-salicylic acid (5-ASA)	[54]
Coated bioadhesive pellets	5-amino-salicylic acid (5-ASA)	[52]
Microgel	5-amino-salicylic acid (5-ASA)	[55]
Matrix tablets	5-amino-salicylic acid (5-ASA)	[56]
Coated tablets	5-amino-salicylic acid (5-ASA)	[57]
Coated microparticles	5-amino-salicylic acid (5-ASA) and curcumin	[58]
Lipid nanoparticles	Dexamethasone	[59]
Beads	Azathioprine	[60]
Microcrystals	Dexamethasone	[61]
Beads and microparticles	Prednisolone and inulin	[62]
swellable hydrogel	pentosan polysulphate (PP)	[63]
Hydrogel	Resveratrol	[64]
Hydrogel	Curcumin	[65]
Hydrogel	6-shogaol	[66]
Hydrogel	siCD98	[67]
Hydrogel	PS-ATNF-α	[68]
Colloidal particles	NK007	[69]
Coated microparticles	AvrA nanoparticles	[70]
Coated pellets	Rutin	[71]
Microspheres	Icariin	[72]
Coated microparticle tablets	Quercetin	[73]
Coated liposomes	Quercetin	[74]
Monolithic tablet	Diamide oxidase and catalase	[75]
Polymer-enzime cojugate	Superoxide dismutase	[76]
Nanoparticles	Berberine	[77]
Coated nanoemulsion	Curcumin	[78]
Coated agglomerates of nanoparticles	Diclofenac sodium	[79]
Nanoparticle	SIGIRR gene	[80]
Microspheres	Ketoprofen and ascorbic acid	[81]

## Data Availability

Not applicable.

## References

[1] Sandberg K., Yarger E., Saeed S. (2020). Updates in diagnosis and management of inflammatory bowel disease. Curr. Probl. Pediatr. Adolesc. Health Care.

[2] Dinesen L.C., Walsh A.J., Protic M.N., Heap G., Cummings F., Warren B.F., George B., Mortensen N.J.M., Travis S.P.L. (2010). The pattern and outcome of acute severe colitis. J. Crohn’s Colitis.

[3] Kaur M., Dalal R.L., Shaffer S., Schwartz D.A., Rubin D.T. (2020). Inpatient Management of Inflammatory Bowel Disease-Related Complications. Clin. Gastroenterol. Hepatol..

[4] Yeshi K., Ruscher R., Hunter L., Daly N.L., Loukas A., Wangchuk P. (2020). Revisiting Inflammatory Bowel Disease: Pathology, Treatments, Challenges and Emerging Therapeutics Including Drug Leads from Natural Products. J. Clin. Med..

[5] Frank D.N., St Amand A.L., Feldman R.A., Boedeker E.C., Harpaz N., Pace N.R. (2007). Molecular-phylogenetic characterization of microbial community imbalances in human inflammatory bowel diseases. Proc. Natl. Acad. Sci. USA.

[6] Roediger W.E.W., Moore J., Babidge W. (1997). Colonic sulfide in pathogenesis and treatment of ulcerative colitis. Dig. Dis. Sci..

[7] Fuss I.J., Neurath M., Boirivant M., Klein J.S., de la Motte C., Strong S.A., Fiocchi C., Strober W. (1996). Disparate CD4+ lamina propria (LP) lymphokine secretion profiles in inflammatory bowel disease. Crohn’s disease LP cells manifest increased secretion of IFN-gamma, whereas ulcerative colitis LP cells manifest increased secretion of IL-5. J. Immunol..

[8] Kolls J.K., Lindén A. (2004). Interleukin-17 family members and inflammation. Immunity.

[9] Alatab S., Sepanlou S.G., Ikuta K., Vahedi H., Bisignano C., Safiri S., Sadeghi A., Nixon M.R., Abdoli A., Abolhassani H. (2020). The global, regional, and national burden of inflammatory bowel disease in 195 countries and territories, 1990–2017: A systematic analysis for the Global Burden of Disease Study 2017. Lancet Gastroenterol. Hepatol..

[10] Kaplan G.G., Ng S.C. (2016). Globalisation of inflammatory bowel disease: Perspectives from the evolution of inflammatory bowel disease in the UK and China. Lancet Gastroenterol. Hepatol..

[11] Aniwan S., Tremaine W.J., Raffals L.E., Kane S.V., Loftus E.V. (2018). Antibiotic Use and New-Onset Inflammatory Bowel Disease in Olmsted County, Minnesota: A Population-Based Case-Control Study. J. Crohn’s Colitis.

[12] Ng S.C., Tang W., Ching J.Y., Wong M., Chow C.M., Hui A.J., Wong T.C., Leung V.K., Tsang S.W., Yu H.H. (2013). Incidence and phenotype of inflammatory bowel disease based on results from the Asia-Pacific Crohn’s and colitis epidemiology study. Gastroenterology.

[13] Yang Y., Owyang C., Wu G.D. (2016). East Meets West: The Increasing Incidence of Inflammatory Bowel Disease in Asia as a Paradigm for Environmental Effects on the Pathogenesis of Immune-Mediated Disease. Gastroenterology.

[14] Vernier-Massouille G., Balde M., Salleron J., Turck D., Dupas J.L., Mouterde O., Merle V., Salomez J.L., Branche J., Marti R. (2008). Natural History of Pediatric Crohn’s Disease: A Population-Based Cohort Study. Gastroenterology.

[15] Lehtinen P., Pasanen K., Kolho K.-L., Auvinen A. (2016). Incidence of Pediatric Inflammatory Bowel Disease in Finland. J. Pediatr. Gastroenterol. Nutr..

[16] Lopez R.N., Appleton L., Gearry R.B., Day A.S. (2018). Rising Incidence of Paediatric Inflammatory Bowel Disease in Canterbury, New Zealand, 1996–2015. J. Pediatr. Gastroenterol. Nutr..

[17] Jose F.A., Garnett E.A., Vittinghoff E., Ferry G.D., Winter H.S., Baldassano R.N., Kirschner B.S., Cohen S.A., Gold B.D., Abramson O. (2009). Development of extraintestinal manifestations in pediatric patients with inflammatory bowel disease. Inflamm. Bowel Dis..

[18] Dotson J.L., Hyams J.S., Markowitz J., LeLeiko N.S., Mack D.R., Evans J.S., Pfefferkorn M.D., Griffiths A.M., Otley A.R., Bousvaros A. (2010). Extraintestinal Manifestations of Pediatric Inflammatory Bowel Disease and Their Relation to Disease Type and Severity. J. Pediatr. Gastroenterol. Nutr..

[19] Jimenez K.M., Gasche C. (2019). Management of Iron Deficiency Anaemia in Inflammatory Bowel Disease. Acta Haematol..

[20] Hashiguchi Y., Muro K., Saito Y., Ito Y., Ajioka Y., Hamaguchi T., Hasegawa K., Hotta K., Ishida H., Ishiguro M. (2020). Japanese Society for Cancer of the Colon and Rectum (JSCCR) guidelines 2019 for the treatment of colorectal cancer. Int. J. Clin. Oncol..

[21] Bishnoi M., Jain A., Hurkat P., Jain S.K. (2016). Chondroitin sulphate: A focus on osteoarthritis. Glycoconj. J..

[22] Volpi N. (2011). Anti-inflammatory activity of chondroitin sulphate: New functions from an old natural macromolecule. Inflammopharmacology.

[23] Du Souich P., García A.G., Vergés J., Montell E. (2009). Immunomodulatory and anti-inflammatory effects of chondroitin sulphate. J. Cell. Mol. Med..

[24] Pumphrey C.Y., Theus A.M., Li S., Parrish R.S., Sanderson R.D. (2002). Neoglycans, Carbodiimide-modified Glycosaminoglycans. Cancer Res..

[25] Borsig L., Wang L., Cavalcante M.C.M., Cardilo-Reis L., Ferreira P.L., Mourão P.A.S., Esko J.D., Pavão M.S.G. (2007). Selectin blocking activity of a fucosylated chondroitin sulfate glycosaminoglycan from sea cucumber: Effect on tumor metastasis and neutrophil recruitment. J. Biol. Chem..

[26] Bergefall K., Trybala E., Johansson M., Uyama T., Naito S., Yamada S., Kitagawa H., Sugahara K., Bergström T. (2005). Chondroitin sulfate characterized by the E-disaccharide unit is a potent inhibitor of herpes simplex virus infectivity and provides the virus binding sites on gro2C cells. J. Biol. Chem..

[27] Kanchana S., Arumugam M., Giji S., Balasubramanian T. (2013). Isolation, characterization and antioxidant activity of hyaluronic acid from marine bivalve mollusc Amussium pleuronectus (Linnaeus, 1758). Bioact. Carbohydr. Diet. Fibre.

[28] Moseley R., Walker M., Waddington R.J., Chen W.Y.J. (2003). Comparison of the antioxidant properties of wound dressing materials-carboxymethylcellulose, hyaluronan benzyl ester and hyaluronan, towards polymorphonuclear leukocyte-derived reactive oxygen species. Biomaterials.

[29] Šoltés L., Mendichi R., Kogan G., Schiller J., Stankovská M., Arnhold J. (2006). Degradative action of reactive oxygen species on hyaluronan. Biomacromolecules.

[30] Greenberg D.D., Stoker A., Kane S., Cockrell M., Cook J.L. (2006). Biochemical effects of two different hyaluronic acid products in a co-culture model of osteoarthritis. Osteoarthr. Cartil..

[31] Petrey A.C., de la Motte C.A. (2019). Hyaluronan in inflammatory bowel disease: Cross-linking inflammation and coagulation. Matrix Biol..

[32] Shah H., Patel R. (2015). Statistical modeling of zaltoprofen loaded biopolymeric nanoparticles: Characterization and anti-inflammatory activity of nanoparticles loaded gel. Int. J. Pharm. Investig..

[33] Kim K.W., Thomas R.L. (2007). Antioxidative activity of chitosans with varying molecular weights. Food Chem..

[34] Kamil J.Y.V.A., Jeon Y.J., Shahidi F. (2002). Antioxidative activity of chitosans of different viscosity in cooked comminuted flesh of herring (*Clupea harengus*). Food Chem..

[35] Hajji S., Younes I., Rinaudo M., Jellouli K., Nasri M. (2015). Characterization and In Vitro Evaluation of Cytotoxicity, Antimicrobial and Antioxidant Activities of Chitosans Extracted from Three Different Marine Sources. Appl. Biochem. Biotechnol..

[36] Azuma K., Osaki T., Minami S., Okamoto Y. (2015). Anticancer and Anti-Inflammatory Properties of Chitin and Chitosan Oligosaccharides. J. Funct. Biomater..

[37] Raafat D., Sahl H.G. (2009). Chitosan and its antimicrobial potential—A critical literature survey. Microb. Biotechnol..

[38] Hajiali H., Summa M., Russo D., Armirotti A., Brunetti V., Bertorelli R., Athanassiou A., Mele E. (2016). Alginate-lavender nanofibers with antibacterial and anti-inflammatory activity to effectively promote burn healing. J. Mater. Chem. B.

[39] Iizima-Mizui N. (1985). Antitumor activity of polysaccharide fractions from the brown seaweed Sargassum kjelimanianum. Kitasato Arch. Exp. Med..

[40] Jeong H.-J., Lee S.-A., Moon P.-D., Na H.-J., Park R.-K., Um J.-Y., Kim H.-M., Hong S.-H. (2006). Alginic acid has anti-anaphylactic effects and inhibits inflammatory cytokine expression via suppression of nuclear factor-κB activation. Clin. Exp. Allergy.

[41] Tomida H., Yasufuku T., Fujii T., Kondo Y., Kai T., Anraku M. (2010). Polysaccharides as potential antioxidative compounds for extended-release matrix tablets. Carbohydr. Res..

[42] Wang P., Jiang X., Jiang Y., Hu X., Mou H., Li M., Guan H. (2007). In vitro antioxidative activities of three marine oligosaccharides. Nat. Prod. Res..

[43] Yoshida T., Hirano A., Wada H., Takahashi K., Hattori M. (2004). Alginic Acid Oligosaccharide Suppresses Th2 Development and IgE Production by Inducing IL-12 Production. Int. Arch. Allergy Immunol..

[44] Younes I., Rinaudo M. (2015). Chitin and chitosan preparation from marine sources. Structure, properties and applications. Mar. Drugs.

[45] Bernkop-Schnürch A., Dünnhaupt S. (2012). Chitosan-based drug delivery systems. Eur. J. Pharm. Biopharm..

[46] Leung T.C.Y., Wong C.K., Xie Y. (2010). Green synthesis of silver nanoparticles using biopolymers, carboxymethylated-curdlan and fucoidan. Mater. Chem. Phys..

[47] Jayakumar R., Menon D., Manzoor K., Nair S.V., Tamura H. (2010). Biomedical applications of chitin and chitosan based nanomaterials—A short review. Carbohydr. Polym..

[48] Lautenschläger C., Schmidt C., Lehr C.M., Fischer D., Stallmach A. (2013). PEG-functionalized microparticles selectively target inflamed mucosa in inflammatory bowel disease. Eur. J. Pharm. Biopharm..

[49] Kootala S., Filho L., Srivastava V., Linderberg V., Moussa A., David L., Trombotto S., Crouzier T. (2018). Reinforcing Mucus Barrier Properties with Low Molar Mass Chitosans. Biomacromolecules.

[50] Xu J., Tam M., Samaei S., Lerouge S., Barralet J., Stevenson M.M., Cerruti M. (2017). Mucoadhesive chitosan hydrogels as rectal drug delivery vessels to treat ulcerative colitis. Acta Biomater..

[51] Bawa P., Choonara Y.E., Du Toit L.C., Kumar P., Ndesendo V.M.K., Meyer L.C.R., Pillay V. (2013). A novel stimuli-synchronized alloy-treated matrix for space-defined gastrointestinal delivery of mesalamine in the Large White pig model. J. Control. Release.

[52] Bautzová T., Rabišková M., Béduneau A., Pellequer Y., Lamprecht A. (2012). Bioadhesive pellets increase local 5-aminosalicylic acid concentration in experimental colitis. Eur. J. Pharm. Biopharm..

[53] Ribeiro L.N.M., Alcântara A.C.S., Darder M., Aranda P., Araújo-Moreira F.M., Ruiz-Hitzky E. (2014). Pectin-coated chitosan-LDH bionanocomposite beads as potential systems for colon-targeted drug delivery. Int. J. Pharm..

[54] Mura C., Nácher A., Merino V., Merino-Sanjuan M., Carda C., Ruiz A., Manconi M., Loy G., Fadda A.M., Diez-Sales O. (2011). N-Succinyl-chitosan systems for 5-aminosalicylic acid colon delivery: In vivo study with TNBS-induced colitis model in rats. Int. J. Pharm..

[55] Chen C., Liu M., Lii S., Gao C., Chen J. (2012). In Vitro Degradation and Drug-Release Properties of Water-Soluble Chitosan Cross-Linked Oxidized Sodium Alginate Core–Shell Microgels. J. Biomater. Sci. Polym. Ed..

[56] Newton A.M.J., Lakshmanan P. (2014). Effect of HPMC—E15 LV premium Polymer on Release Profile and Compression Characteristics of Chitosan/ Pectin Colon Targeted Mesalamine Matrix Tablets and in vitro Study on Effect of pH Impact on the Drug Release Profile. Recent Pat. Drug Deliv. Formul..

[57] Singh K., Suri R., Tiwary A.K., Rana V. (2013). Exploiting the synergistic effect of chitosan-EDTA conjugate with MSA for the early recovery from colitis. Int. J. Biol. Macromol..

[58] Duan H., Lü S., Gao C., Bai X., Qin H., Wei Y., Wu X., Liu M. (2016). Mucoadhesive microparticulates based on polysaccharide for target dual drug delivery of 5-aminosalicylic acid and curcumin to inflamed colon. Colloids Surf. B Biointerfaces.

[59] Chen S.-Q., Song Y.-Q., Wang C., Tao S., Yu F.-Y., Lou H.-Y., Hu F.-Q., Yuan H. (2020). Chitosan-modified lipid nanodrug delivery system for the targeted and responsive treatment of ulcerative colitis. Carbohydr. Polym..

[60] Helmy A.M., Elsabahy M., Soliman G.M., Mahmoud M.A., Ibrahim E.A. (2017). Development and in vivo evaluation of chitosan beads for the colonic delivery of azathioprine for treatment of inflammatory bowel disease. Eur. J. Pharm. Sci..

[61] Oshi M.A., Naeem M., Bae J., Kim J., Lee J., Hasan N., Kim W., Im E., Jung Y., Yoo J.W. (2018). Colon-targeted dexamethasone microcrystals with pH-sensitive chitosan/alginate/Eudragit S multilayers for the treatment of inflammatory bowel disease. Carbohydr. Polym..

[62] Araujo V., Gamboa A., Caro N., Abugoch L., Gotteland M., Valenzuela F., Merchant H.A., Basit A.W., Tapia C. (2013). Release of prednisolone and inulin from a new calcium-alginate chitosan-coated matrix system for colonic delivery. J. Pharm. Sci..

[63] Shah H.K., Conkie J.A., Tait R.C., Johnson J.R., Wilson C.G. (2011). A novel, biodegradable and reversible polyelectrolyte platform for topical-colonic delivery of pentosan polysulphate. Int. J. Pharm..

[64] Iglesias N., Galbis E., Díaz-Blanco M.J., Lucas R., Benito E., De-Paz M.V. (2019). Nanostructured Chitosan-based biomaterials for sustained and colon-specific resveratrol release. Int. J. Mol. Sci..

[65] Zhang X., Ma Y., Ma L., Zu M., Song H., Xiao B. (2019). Oral administration of chondroitin sulfate-functionalized nanoparticles for colonic macrophage-targeted drug delivery. Carbohydr. Polym..

[66] Zhang M., Xu C., Liu D., Han M.K., Wang L., Merlin D. (2018). Oral Delivery of Nanoparticles Loaded With Ginger Active Compound, 6-Shogaol, Attenuates Ulcerative Colitis and Promotes Wound Healing in a Murine Model of Ulcerative Colitis. J. Crohn’s Colitis.

[67] Xiao B., Laroui H., Viennois E., Ayyadurai S., Charania M.A., Zhang Y., Zhang Z., Baker M.T., Zhang B., Gewirtz A.T. (2014). Nanoparticles with surface antibody against CD98 and carrying CD98 small interfering RNA reduce colitis in mice. Gastroenterology.

[68] Duan B., Li M., Sun Y., Zou S., Xu X. (2019). Orally Delivered Antisense Oligodeoxyribonucleotides of TNF-α via Polysaccharide-Based Nanocomposites Targeting Intestinal Inflammation. Adv. Healthc. Mater..

[69] Chen S., Wang J., Cheng H., Guo W., Yu M., Zhao Q., Wu Z., Zhao L., Yin Z., Hong Z. (2015). Targeted Delivery of NK007 to Macrophages to Treat Colitis. J. Pharm. Sci..

[70] Ling K., Wu H., Neish A.S., Champion J.A. (2019). Alginate/chitosan microparticles for gastric passage and intestinal release of therapeutic protein nanoparticles. J. Control. Release.

[71] Rabišková M., Bautzová T., Gajdziok J., Dvořáčková K., Lamprecht A., Pellequer Y., Spilková J. (2012). Coated chitosan pellets containing rutin intended for the treatment of inflammatory bowel disease: In vitro characteristics and in vivo evaluation. Int. J. Pharm..

[72] Wang Q.S., Wang G.F., Zhou J., Gao L.N., Cui Y.L. (2016). Colon targeted oral drug delivery system based on alginate-chitosan microspheres loaded with icariin in the treatment of ulcerative colitis. Int. J. Pharm..

[73] Caddeo C., Nácher A., Díez-Sales O., Merino-Sanjuán M., Fadda A.M., Manconi M. (2014). Chitosan-xanthan gum microparticle-based oral tablet for colon-targeted and sustained delivery of quercetin. J. Microencapsul..

[74] Caddeo C., Díez-Sales O., Pons R., Carbone C., Ennas G., Puglisi G., Fadda A.M., Manconi M. (2016). Cross-linked chitosan/liposome hybrid system for the intestinal delivery of quercetin. J. Colloid Interface Sci..

[75] Calinescu C., Mondovi B., Federico R., Ispas-Szabo P., Mateescu M.A. (2012). Carboxymethyl starch: Chitosan monolithic matrices containing diamine oxidase and catalase for intestinal delivery. Int. J. Pharm..

[76] Zhao N., Feng Z., Shao M., Cao J., Wang F., Liu C. (2017). Stability profiles and therapeutic effect of cu/zn superoxide dismutase chemically coupled to o-quaternary chitosan derivatives against dextran sodium sulfate-induced colitis. Int. J. Mol. Sci..

[77] Wu S.J., Don T.M., Lin C.W., Mi F.L. (2014). Delivery of berberine using chitosan/fucoidan-taurine conjugate nanoparticles for treatment of defective intestinal epithelial tight junction barrier. Mar. Drugs.

[78] Langella A., Calcagno V., De Gregorio V., Urciuolo F., Imparato G., Vecchione R., Netti P.A. (2018). In vitro study of intestinal epithelial interaction with engineered oil in water nanoemulsions conveying curcumin. Colloids Surf. B Biointerfaces.

[79] Huanbutta K., Sriamornsak P., Luangtana-Anan M., Limmatvapirat S., Puttipipatkhachorn S., Lim L.Y., Terada K., Nunthanid J. (2013). Application of multiple stepwise spinning disk processing for the synthesis of poly(methyl acrylates) coated chitosan-diclofenac sodium nanoparticles for colonic drug delivery. Eur. J. Pharm. Sci..

[80] Liu J., Chen Y., Liu D., Liu W., Hu S., Zhou N., Xie Y. (2017). Ectopic expression of SIGIRR in the colon ameliorates colitis in mice by downregulating TLR4/NF-κB overactivation. Immunol. Lett..

[81] Maestrelli F., Zerrouk N., Cirri M., Mura P. (2015). Comparative evaluation of polymeric and waxy microspheres for combined colon delivery of ascorbic acid and ketoprofen. Int. J. Pharm..

[82] Naveed M., Phil L., Sohail M., Hasnat M., Baig M.M.F.A., Ihsan A.U., Shumzaid M., Kakar M.U., Mehmood Khan T., Akabar M.D. (2019). Chitosan oligosaccharide (COS): An overview. Int. J. Biol. Macromol..

[83] Muanprasat C., Wongkrasant P., Satitsri S., Moonwiriyakit A., Pongkorpsakol P., Mattaveewong T., Pichyangkura R., Chatsudthipong V. (2015). Activation of AMPK by chitosan oligosaccharide in intestinal epithelial cells: Mechanism of action and potential applications in intestinal disorders. Biochem. Pharmacol..

[84] Huang B., Xiao D., Tan B., Xiao H., Wang J., Yin J., Duan J., Huang R., Yang C., Yin Y. (2016). Chitosan Oligosaccharide Reduces Intestinal Inflammation That Involves Calcium-Sensing Receptor (CaSR) Activation in Lipopolysaccharide (LPS)-Challenged Piglets. J. Agric. Food Chem..

[85] Mattaveewong T., Wongkrasant P., Chanchai S., Pichyangkura R., Chatsudthipong V., Muanprasat C. (2016). Chitosan oligosaccharide suppresses tumor progression in a mouse model of colitis-associated colorectal cancer through AMPK activation and suppression of NF-κB and mTOR signaling. Carbohydr. Polym..

[86] Yang J.W., Tian G., Chen D.W., Yao Y., He J., Zheng P., Mao X.B., Yu J., Huang Z.Q., Yu B. (2018). Involvement of PKA signalling in anti-inflammatory effects of chitosan oligosaccharides in IPEC-J2 porcine epithelial cells. J. Anim. Physiol. Anim. Nutr..

[87] Denost Q., Adam J.P., Pontallier A., Montembault A., Bareille R., Siadous R., Delmond S., Rullier E., David L., Bordenave L. (2015). Colorectal tissue engineering: A comparative study between porcine small intestinal submucosa (SIS) and chitosan hydrogel patches. Surgery.

[88] Quentin D., Arnaud P., Etienne B., Reine B., Robin S., Marlene D., Samantha D., Laurent D., Laurence B. (2017). Colorectal wall regeneration resulting from the association of chitosan hydrogel and stromal vascular fraction from adipose tissue. J. Biomed. Mater. Res. Part A.

[89] Fraser J.R.E., Laurent T.C., Laurent U.B.G. (1997). Hyaluronan: Its nature, distribution, functions and turnover. J. Intern. Med..

[90] Lindahl U., Couchman J., Kimata K., Esko J.D. (2015). Proteoglycans and Sulfated Glycosaminoglycans.

[91] Burdick J.A., Prestwich G.D. (2011). Hyaluronic acid hydrogels for biomedical applications. Adv. Mater..

[92] Toole B.P. (2001). Hyaluronan in morphogenesis. Semin. Cell Dev. Biol..

[93] Kogan G., Šoltés L., Stern R., Gemeiner P. (2007). Hyaluronic acid: A natural biopolymer with a broad range of biomedical and industrial applications. Biotechnol. Lett..

[94] Giji S., Arumugam M. (2014). Isolation and characterization of hyaluronic acid from marine organisms. Advances in Food and Nutrition Research.

[95] Vázquez J.A., Rodríguez-Amado I., Montemayor M.I., Fraguas J., Del González M.P., Murado M.A. (2013). Chondroitin sulfate, hyaluronic acid and chitin/chitosan production using marine waste sources: Characteristics, applications and eco-friendly processes: A review. Mar. Drugs.

[96] Abdallah M.M., Fernández N., Matias A.A., do Rosario Bronze M. (2020). Hyaluronic acid and Chondroitin sulfate from marine and terrestrial sources: Extraction and purification methods. Carbohydr. Polym..

[97] Collins M.N., Birkinshaw C. (2013). Hyaluronic acid based scaffolds for tissue engineering—A review. Carbohydr. Polym..

[98] Passi A., Vigetti D. (2019). Hyaluronan as tunable drug delivery system. Adv. Drug Deliv. Rev..

[99] Fiorino G., Gilardi D., Naccarato P., Sociale O.R., Danese S. (2014). Safety and efficacy of sodium hyaluronate (IBD98E) in the induction of clinical and endoscopic remission in subjects with distal ulcerative colitis. Dig. Liver Dis..

[100] Sammarco G., Shalaby M., Elangovan S., Petti L., Roda G., Restelli S., Arena V., Ungaro F., Fiorino G., Day A.J. (2019). Hyaluronan Accelerates Intestinal Mucosal Healing through Interaction with TSG-6. Cells.

[101] Chiu C.-T., Kuo S.-N., Hung S.-W., Yang C.-Y. (2017). Combined Treatment with Hyaluronic Acid and Mesalamine Protects Rats from Inflammatory Bowel Disease Induced by Intracolonic Administration of Trinitrobenzenesulfonic Acid. Molecules.

[102] Narayanaswamy R., Torchilin V.P. (2019). Hydrogels and Their Applications in Targeted Drug Delivery. Molecules.

[103] Luo J.W., Liu C., Wu J.H., Lin L.X., Fan H.M., Zhao D.H., Zhuang Y.Q., Sun Y.L. (2019). In situ injectable hyaluronic acid/gelatin hydrogel for hemorrhage control. Mater. Sci. Eng. C.

[104] Aprodu A., Mantaj J., Raimi-Abraham B., Vllasaliu D. (2019). Evaluation of a Methylcellulose and Hyaluronic Acid Hydrogel as a Vehicle for Rectal Delivery of Biologics. Pharmaceutics.

[105] Fattahi F.S., Khoddami A., Avinc O. (2020). Sustainable, Renewable, and Biodegradable Poly(Lactic Acid) Fibers and Their Latest Developments in the Last Decade.

[106] Makkar S.K., Riehl T.E., Stenson W.F. (2017). Blocking Hyaluronic Acid Binding to TLR4 Results in Decreased Growth of Colon Cancer and Increased Sensitivity to Radiation. Gastroenterology.

[107] Mármol I., Sánchez-de-Diego C., Pradilla Dieste A., Cerrada E., Rodriguez Yoldi M. (2017). Colorectal Carcinoma: A General Overview and Future Perspectives in Colorectal Cancer. Int. J. Mol. Sci..

[108] Qu D., Wang L., Huo M., Song W., Lau C.-W., Xu J., Xu A., Yao X., Chiu J.-J., Tian X.Y. (2020). Focal TLR4 activation mediates disturbed flow-induced endothelial inflammation. Cardiovasc. Res..

[109] Wachsmann P., Lamprecht A. (2012). Polymeric nanoparticles for the selective therapy of inflammatory bowel disease. Methods in Enzymology.

[110] Xiao B., Han M.K., Viennois E., Wang L., Zhang M., Si X., Merlin D. (2015). Hyaluronic acid-functionalized polymeric nanoparticles for colon cancer-targeted combination chemotherapy. Nanoscale.

[111] Si X.Y., Merlin D., Xiao B. (2016). Recent advances in orally administered cell-specific nanotherapeutics for inflammatory bowel disease. World J. Gastroenterol..

[112] Lautenschläger C., Schmidt C., Lange K., Stallmach A. (2015). Drug-Delivery-Strategien zur gezielten Behandlung von chronisch-entzündlichen Darmerkrankungen. Z. Gastroenterol..

[113] Xiao B., Xu Z., Viennois E., Zhang Y., Zhang Z., Zhang M., Han M.K., Kang Y., Merlin D. (2017). Orally Targeted Delivery of Tripeptide KPV via Hyaluronic Acid-Functionalized Nanoparticles Efficiently Alleviates Ulcerative Colitis. Mol. Ther..

[114] Xiao B., Zhang Z., Viennois E., Kang Y., Zhang M., Han M.K., Chen J., Merlin D. (2016). Combination Therapy for Ulcerative Colitis: Orally Targeted Nanoparticles Prevent Mucosal Damage and Relieve Inflammation. Theranostics.

[115] Farkas S., Hornung M., Sattler C., Anthuber M., Gunthert U., Herfarth H., Schlitt H.J., Geissler E.K., Wittig B.M. (2005). Short-term treatment with anti-CD44v7 antibody, but not CD44v4, restores the gut mucosa in established chronic dextran sulphate sodium (DSS)-induced colitis in mice. Clin. Exp. Immunol..

[116] Hankard G.F., Cezard J.P., Aigrain Y., Navarro J., Peuchmaur M. (1998). CD44 variant expression in inflammatory colonic mucosa is not disease specific but associated with increased crypt cell proliferation. Histopathology.

[117] Dreaden E.C., Morton S.W., Shopsowitz K.E., Choi J.H., Deng Z.J., Cho N.J., Hammond P.T. (2014). Bimodal tumor-targeting from microenvironment responsive hyaluronan layer-by-layer (LbL) nanoparticles. ACS Nano.

[118] Vafaei S.Y., Esmaeili M., Amini M., Atyabi F., Ostad S.N., Dinarvand R. (2016). Self assembled hyaluronic acid nanoparticles as a potential carrier for targeting the inflamed intestinal mucosa. Carbohydr. Polym..

[119] Li W., Li Y., Liu Z., Kerdsakundee N., Zhang M., Zhang F., Liu X., Bauleth-Ramos T., Lian W., Mäkilä E. (2018). Hierarchical structured and programmed vehicles deliver drugs locally to inflamed sites of intestine. Biomaterials.

[120] Sugahara K., Kitagawa H. (2000). Recent advances in the study of the biosynthesis and functions of sulfated glycosaminoglycans. Curr. Opin. Struct. Biol..

[121] Sugahara K., Mikami T., Uyama T., Mizuguchi S., Nomura K., Kitagawa H. (2003). Recent advances in the structural biology of chondroitin sulfate and dermatan sulfate. Curr. Opin. Struct. Biol..

[122] Kusche-Gullberg M., Kjellén L. (2003). Sulfotransferases in glycosaminoglycan biosynthesis. Curr. Opin. Struct. Biol..

[123] Malavaki C., Mizumoto S., Karamanos N., Sugahara K. (2008). Recent advances in the structural study of functional chondroitin sulfate and dermatan sulfate in health and disease. Connect. Tissue Res..

[124] Cesar A.L.A., Abrantes F.A., Farah L., Castilho R.O., Cardoso V., Fernandes S.O., Araújo I.D., Faraco A.A.G. (2018). New mesalamine polymeric conjugate for controlled release: Preparation, characterization and biodistribution study. Eur. J. Pharm. Sci..

[125] Onishi H., Ikeuchi-Takahashi Y., Kawano K., Hattori Y. (2019). Preparation of chondroitin sulfate-glycyl-prednisolone conjugate nanogel and its efficacy in rats with ulcerative colitis. Biol. Pharm. Bull..

[126] Gou S., Huang Y., Wan Y., Ma Y., Zhou X., Tong X., Huang J., Kang Y., Pan G., Dai F. (2019). Multi-bioresponsive silk fibroin-based nanoparticles with on-demand cytoplasmic drug release capacity for CD44-targeted alleviation of ulcerative colitis. Biomaterials.

[127] Linares P.M., Chaparro M., Algaba A., Román M., Moreno Arza I., Abad Santos F., Ochoa D., Guerra I., Bermejo F., Gisbert J.P. (2015). Effect of Chondroitin Sulphate on Pro-Inflammatory Mediators and Disease Activity in Patients with Inflammatory Bowel Disease. Digestion.

[128] Ching S.H., Bansal N., Bhandari B. (2017). Alginate gel particles–A review of production techniques and physical properties. Crit. Rev. Food Sci. Nutr..

[129] Vera J., Castro J., Gonzalez A., Moenne A. (2011). Seaweed Polysaccharides and Derived Oligosaccharides Stimulate Defense Responses and Protection Against Pathogens in Plants. Mar. Drugs.

[130] Aderibigbe B.A., Buyana B. (2018). Alginate in Wound Dressings. Pharmaceutics.

[131] Lee K.Y., Mooney D.J. (2012). Alginate: Properties and biomedical applications. Prog. Polym. Sci..

[132] García-Ríos V., Ríos-Leal E., Robledo D., Freile-Pelegrin Y. (2012). Polysaccharides composition from tropical brown seaweeds. Phycol. Res..

[133] Paques J.P., van der Linden E., van Rijn C.J.M., Sagis L.M.C. (2014). Preparation methods of alginate nanoparticles. Adv. Colloid Interface Sci..

[134] Pawar S.N., Edgar K.J. (2012). Alginate derivatization: A review of chemistry, properties and applications. Biomaterials.

[135] Wong T.W. (2011). Alginate graft copolymers and alginate-co-excipient physical mixture in oral drug delivery. J. Pharm. Pharm..

[136] Krishnaiah Y.S.R., Khan M.A. (2012). Strategies of targeting oral drug delivery systems to the colon and their potential use for the treatment of colorectal cancer. Pharm. Dev. Technol..

[137] Samak Y.O., El Massik M., Coombes A.G.A. (2017). A Comparison of Aerosolization and Homogenization Techniques for Production of Alginate Microparticles for Delivery of Corticosteroids to the Colon. J. Pharm. Sci..

[138] Samak Y.O., Santhanes D., El-Massik M.A., Coombes A.G.A. (2019). Formulation strategies for achieving high delivery efficiency of thymoquinone-containing Nigella sativa extract to the colon based on oral alginate microcapsules for treatment of inflammatory bowel disease. J. Microencapsul..

[139] You Y.C., Dong L.Y., Dong K., Xu W., Yan Y., Zhang L., Wang K., Xing F.J. (2015). In vitro and in vivo application of pH-sensitive colon-targeting polysaccharide hydrogel used for ulcerative colitis therapy. Carbohydr. Polym..

[140] Md Ramli S.H., Wong T.W., Naharudin I., Bose A. (2016). Coatless alginate pellets as sustained-release drug carrier for inflammatory bowel disease treatment. Carbohydr. Polym..

